# Do medical students want to learn about global health?

**DOI:** 10.3402/gha.v7.23943

**Published:** 2014-05-16

**Authors:** Anya Göpfert, Hussein Mohamedbhai, Josko Mise, Anne Driessen, Ambreen Shakil, Ann Fitzmaurice, Wendy Graham

**Affiliations:** 1 International Federation of Medical Students Associations, Ferney-Voltaire, France; 2 School of Medicine and Dentistry, Newcastle University, Newcastle Upon Tyne, UK; 3 Royal Berkshire Hospital, Reading, UK; 4 School of Medicine, University of Zagreb, Zagreb, Croatia; 5 School of Medicine, Leiden University, Leiden, The Netherlands; 6 School of Medicine and Dentistry, University of Aberdeen, Aberdeen, UK

**Keywords:** global, maternal, child, health, student, curriculum, medical education

## Abstract

**Background:**

One objective of the United Nations Global Strategy for Women's and Children's Health relates to ensuring a sufficiently skilled workforce. To prepare future healthcare professionals for their role in the 21st century as members of this workforce, awareness of global health is essential, but few studies have explored student perspectives on such education. The main objectives of this study were to establish the views of medical students on learning about women's and children's health in low-income countries, to identify the nature and extent of learning already experienced, and to assess the demand for such learning.

**Design:**

A questionnaire survey was conducted at three meetings of the International Federation of Medical Students Associations (IFMSA). Questionnaires were distributed to 500 participants from 75 countries and 336 medical schools, and 492 usable questionnaires were returned. Data were analysed using SPSS Version 20 and statistical analysis was undertaken using Fisher's exact test.

**Results:**

There were 492 questionnaires included in the analysis. Forty-eight per cent of participants were from low–middle income countries and 52% were from high-income countries. Less than half (43%) of the respondents had received some teaching on women's and children's health in low-income countries. Teaching received was primarily (96%) through lectures in the second year of study. Ninety-one per cent of respondents thought such teaching would be important and stated that group work (66%) would be the preferred method. In total, only 14% thought they had received sufficient teaching on global health and on women's and children's health in low-income countries.

**Conclusions:**

This study has revealed a high demand among medical students for global health teaching, particularly on women's and children's health in low-income countries. The timing and methods of existing teaching on these topics does not match that desired by medical students. To help address this gap, a collaborative approach is proposed which includes students’ views in the processes for revitalising medical curricula to meet the needs of the 21st century.

In September 2010, the Global Strategy for Women's and Children's Health was launched by the United Nations (1). The main aim of the Global Strategy is to accelerate progress in improving women's and children's health through enhanced financing, strengthened policy, and integrated service delivery. Within the Global Strategy there are six priority areas, one of which is ‘stronger health systems with sufficient skilled health workers at their core’.

In the United Kingdom, since the introduction of the Tomorrow's Doctors policy statement (2), student perspectives on their own education and experiences of teaching have started to form a key component of the development and reform of medical curricula. Placing the student at the centre of learning outcomes implies they should also have a valid and relevant role in curriculum content (3).

The International Federation of Medical Students Associations (IFMSA) is an umbrella organisation which provides a forum for medical students from 116 countries, spanning six continents. As an organisation representing 1.2 million students, the IFMSA recognised the important contribution that it could make to improve the health of women and children. In September 2011, the IFMSA made a commitment to the UN Global Strategy by agreeing to assess the exposure of medical students to education regarding women's and children's health globally and to global health education more broadly.

Global health has been defined as

an area for study, research, and practice that places a priority on improving health and achieving equity in health for all people worldwide. Global health emphasises transnational health issues, determinants and solutions; involves many disciplines within and beyond the health sciences and promotes interdisciplinary collaboration; and is a synthesis of population-based prevention with individual-level clinical care. (4)

The main objectives of this study were:

1. To establish the views of undergraduate medical students about learning on women's and children's health in low-income countries;
2. To identify the nature and extent of education on global health and interdisciplinary learning that participants have experienced, and their satisfaction with this;
3. To assess the demand for learning on women's and children's health in low-income countries amongst the student body.

## Methods

An initial review of the literature was conducted concerning student perspectives of education on global health and maternal and child health. Only a few relevant studies could be identified, and these had small sample populations from medical schools within the same country or within the same geographical location (5–11). No published studies exploring student experiences of education on global health across a large range of countries could be found.

### Questionnaire development

To secure the views of a broad representative group of medical students from across low-, middle-, and high-income countries (HIC), a questionnaire survey was identified as the optimal method. Relevant articles on medical student learning and on global health teaching identified by the literature review were used to inform the selection of question topics and definitions to be used in the survey. A list of potential questions was then reviewed through consultations amongst the working group (comprising IFMSA students from different parts of the world), and a small steering group (comprising a team of academics at the University of Aberdeen). The final questions used in the questionnaire were developed through consecutive pilot surveys. The two meetings used for piloting were the IFMSA General Assembly in Accra Ghana (March 2012) and the Prague European Regional Meeting (April 2012). The experience and findings from the two pilots were used by the investigator group to revise the content of the questionnaire and the final approach to be used to distribute the schedule. The questions were all posed in English and a copy of the final schedule can be seen in Supplementary file.

### Sample

IFMSA General Assembly Conferences frequently attract around 1,000 students from the 116 member countries. The attendees are either medical students or recently qualified doctors, who study or studied in a wide range of low- and middle-income countries (LMIC) and HIC.

A convenience sample was used comprising student participants attending the IFMSA General Assembly Conference in Mumbai in August 2012. Permission to conduct the survey was provided by the Executive Board on behalf of the IFMSA members, as described further below. Paper questionnaires were distributed among the students by the authors of the study, and completed schedules were returned to various collection points at the conference venue.

### Data analysis

The data from all the questionnaires were double-entered into an SPSS database at the University of Aberdeen. Prior to analysis, the data were checked for inconsistencies and cross-reference made to the original questionnaires to inform corrections. The data were analysed using SPSS version 20, producing simple frequencies and crosstabs, and statistically significant differences were tested for using Fisher's exact test. The latter was used to identify associations between the respondents’ answers and student sub-groups. The primary analysis presented in this paper is based on the income grouping of the country of the respondent's medical school, broadly divided into ‘developed’ (HIC) and ‘developing’ (LMIC).

### Ethical considerations

The study was conducted as an internal audit of student experiences and views of education on global health. Student members of the IFMSA surveyed their peers within the organisation and permission to conduct the questionnaire survey at IFMSA meetings was granted by the IFMSA Executive Board. All participants received an explanation of the study and how the data would be used. Participation was voluntary and participants retained the right to withdraw at any time. All questionnaires were completed anonymously.

##  Results

A total of 500 questionnaires were distributed, of which 492 were returned and entered into the analysis. Eight questionnaires were excluded owing to incomplete entries. A total of 40.6% of the sample (199 participants) was male, and the median age of participants was 22 years. The participants came from 336 medical schools across 75 countries, with 48% of respondents from LMIC and 52% from HIC.

### Previous teaching received

Overall 43% of participants had received teaching on women's and children's health in low-income countries. There was a significant difference among this finding, with 57% of those from LMIC receiving teaching compared to 30% of those from HIC (*p*<0.001), as indicated in Table 1. Of the respondents who had received teaching on women's and children's health, 84% had also received teaching on other global health topics.

**Table 1:** Key results on learning on global health in LMIC and HIC

Question topic	Response categories	Total (%)	LMIC (%)	HIC (%)	*p* value (Fisher's exact test)
Previous teaching received	Received teaching on women's and children's health in LMIC	212 (43)	135 (57)	77 (30)	<0.001
	Teaching on other aspects of global health	177 (84)	109 (81)	68 (88)	0.153
Content of teaching	Measuring global health	168 (80)	112 (84)	56 (73)	0.06
	Burden	172 (82)	114 (85)	58 (75)	0.079
	Broad determinants	146 (69)	96 (72)	50 (65)	0.31
	Interventions and strategies	129 (61)	92 (69)	37 (48)	0.003
	Health care and public health systems	121 (57)	89 (66.4)	32 (42)	<0.001
	Global co-operation and initiatives	93 (44)	59 (44)	34 (44)	0.986
	Rights, equity and social justice	105 (50)	69 (52)	36 (47)	0.507
Timing of teaching	Teaching in 1st year of studies – women's and children's health in LMIC	56 (27)	27 (20)	29 (38)	0.004
	Teaching in 1st year of studies – global health	56 (32)	28 (26)	28 (41)	0.031
Mode of delivery*	Lectures	203 (96)	128 (96)	75 (97)	0.491
	Tutorials	61 (29)	48 (36)	13 (17)	0.003
	Group work	56 (27)	36 (27)	20 (26)	0.888
	Self-directed learning	47 (22)	34 (25)	13 (17)	0.154
	Student elective project	33 (16)	19 (14)	14 (18)	0.441
	Student exchange scheme	9 (4)	4 (3)	5 (7)	0.225
	Other	14 (7)	9 (7)	5 (7)	0.95

* Participants could select more than 1 answer. LMIC=low–middle income countries; HIC=high-income countries.

### Timing of global health teaching

**Fig. 1:**
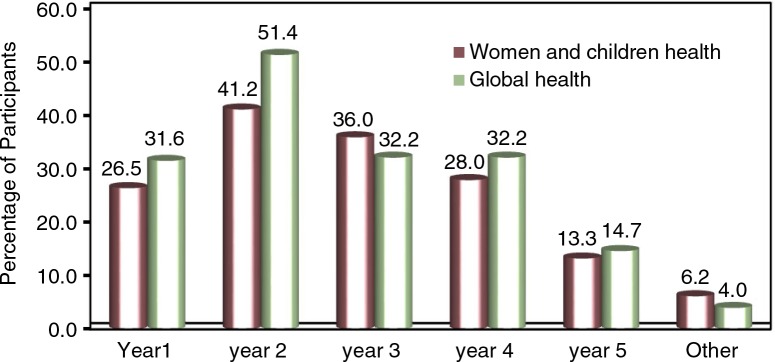
Year of study in which teaching was received.

Sixty-two per cent of participants reported receiving global health teaching during only 1 year of their medical studies. This teaching was undertaken in the first and second years of the medical course in 83% of cases, as seen in Fig. 1. Students from HIC were significantly more likely to receive global health teaching in the first year of their degree, on both women's and children's health and global health more broadly. There was no statistically significant difference between LMIC and HIC respondents with regards to teaching in other years of medical school.

### Methods of teaching

The majority of respondents reported lectures as the method of teaching. Significantly more participants from LMIC had tutorials on global health topics, as also seen in Table 1. Of the 492 respondents, only 33% felt that the time spent was sufficient for their learning needs.

### Content of learning

The majority of students had teaching on topics related to measuring the health of women and children (82%), to the burden of mortality and morbidity (80%), and to the broad determinants (69%). Significantly more participants from LMIC had received teaching on interventions and strategies, and on healthcare and public health systems (Table 1).

Less than half of the respondents (41%) had been exposed to inter-disciplinary learning. There was a significant difference in responses between students from LMIC and HIC, with those from the HIC (53%) more likely to report some exposure to interdisciplinary learning, in comparison to 15% from LMIC.

### Demand for future learning

Table 2 details participants’ responses with regard to demands for future learning. Of the 492 respondents, 91% considered it important to receive some teaching on women's and children's health in low-income countries and 94% on global health in general. The majority (71%) indicated that the learning should be compulsory and available in all medical schools. Most respondents thought that learning would be better achieved through group work (66%), whilst delivery through lectures (53%) and student exchange schemes (50%) were also considered important. Significantly more of the respondents from HIC reported that global health education should be optional compared to respondents from LMIC (*p*<0.001). There was no statistical significant difference between HIC and LMIC respondents with regard to whether the teaching should be compulsory.

**Table 2:** Demand for future learning

Question topic	Response categories	Total (%)	LMIC (%)	HIC (%)	*p* value (Fisher's exact test)
Important for student to receive some	Yes	449 (91)	228 (97.0)	221 (87.0)	<0.001
teaching or learning opportunities	No	7 (2)	2 (1)	5 (2)	
on women's and children's health in LMIC	Not sure	33 (7)	5 (2)	28 (11)	
Important for students to learn about	Yes	464 (94)	225 (97)	239 (94)	0.224
global health in general	No	6 (1)	1 (0.4)	5 (2)	
	Not sure	18 (4)	7 (3)	11 (4)	
Teaching should be optional/compulsory	Optional	117 (24)	55 (24)	62 (24)	<0.001
	Compulsory	348 (71)	170 (73)	178 (70)	
	Not sure	23 (5)	8 (3)	15 (6)	
Learning be made available in all or	All medical schools	478 (97)	227 (97)	251 (99)	0.098
some medical schools	Only medical schools in LMIC	11 (2)	8 (3)	3 (1)	
Learning on women's and children's	Lectures	258 (53)	114 (49)	144 (57)	0.078
health best provided by:	Tutorials	167 (34)	78 (33)	89 (35)	0.690
	Group work	323 (66)	163 (70)	160 (63)	0.123
	Self-directed learning	95 (19)	47 (20)	48 (19)	0.742
	Student elective project	193 (39)	91 (39)	102 (40)	0.773
	Student exchange scheme	245 (50)	109 (46)	136 (53)	0.124
	Other	19 (4)	10 (4)	9 (4)	0.678

LMIC =low–middle income countries; HIC= high-income countries.

Of those who had received teaching, 46% felt that the time given was insufficient for their learning needs; a further 21% were not sure if time was sufficient or not. There was a significant association between the gender of the respondents and whether they had reported that the learning time was sufficient (52% of female respondents as compared to 39% of male respondents thought that time was insufficient).

## Discussion

The findings of this study resonate with wider debates in the field of education for global health improvement. Fundamental to healthcare delivery and optimal health is an adequate supply of sufficiently skilled health workers, as stated in the Global Strategy of Women's and Children's Health (1). However, deficiencies within pre-service education of healthcare professionals do exist (7), and this has led to a gap between graduates’ knowledge, skills and competencies and the needs of patients and populations (6). In 2010, Frenk and colleagues assessed education systems for health professionals and argued that these are not responsive to either present or likely future challenges to global health (8).

The results of our study provide further insights into the gaps in medical education, based on students’ own perspectives on adequacy of learning. An overwhelming majority (91%) of the students surveyed thought that global health and women's and children's health in particular should be a component of medical curricula in all medical schools, in both LMIC and HIC. However, this demand is not matched by the teaching experiences of these students. The results of this study also highlight that a minority of students received interdisciplinary teaching, with the lowest exposure in LMIC. Although the role of interdisciplinary learning in health professional education is not universally accepted amongst medical educationalists, our survey findings reveal that adoption has already taken place in some medical schools. The incorporation of an interdisciplinary approach requires effective institutional collaboration, which some authors (8) suggest could be a significant obstacle to progress in implementing such learning.

Interestingly, there have been studies on the inclusion of global health in nursing school curricula, and in relation to the competencies which students should achieve through their undergraduate degrees programmes (7, 8). Several competencies identified for nursing students, in fact, overlap and concur with our findings for medical students. The focus on the global burden of disease, particularly morbidity and mortality, as a key educational component of curricula was noted in both our survey and in these nursing studies (9, 11).

Another recent study, published in 2012 by Rowson and colleagues (5) examined the curricula of one university and found that participants highlighted social determinants of health as key components of medical teaching. This finding is similar to the views of participants in our study. The results from Rowson et al. also emphasised the importance of leadership and policy development as important competencies to be included in curricula, but these are issues which did not feature in our findings. A further study, of Italian medical schools (12), found that global health learning increased in popularity among students between 2007 and 2010 – a finding which concurs with the high demand among participants in our study.

Our study also echoes the findings of other research, which suggests that education on global health is often poorly integrated into undergraduate medical curricula. There are, nevertheless, some promising signs that student perspectives are starting to be included in curriculum development (5), and the current study provides further evidence on why such developments are important and relevant.

However, there are a number of limitations to our study, which must be acknowledged. Firstly, despite efforts to minimise selection bias among respondents by working through a student organisation with global membership, this is still likely to have had an impact upon the generalisability of the study findings. The students who attended the IFMSA Mumbai conference where the questionnaire was distributed are a subset of the overall membership and by virtue of the event being held in a LMIC, may be more favourably disposed to global health issues. Second, the questionnaire was only offered in the English language, and this may have disadvantaged students for whom English is not their first language, dissuading them from participating in the survey or leading to misunderstanding specific questions.

This remains, however, the largest published study to explore medical student views and experiences of education on global health and on women's and children's health, drawn from 75 countries and 336 medical schools. Further studies involving students from other healthcare disciplines would add to our findings and so help build-up a comprehensive assessment of exposure and demand for such learning across the entire future healthcare workforce.

Responding to student demands and needs ultimately requires a collaborative approach between key members of teaching faculties, professional bodies, and national authorities and governments with ultimate responsibility for ensuring a functional health system, including a fit-for-purpose workforce. The IFMSA is well-placed to play a useful role in this collaborative effort. By working at an international level with key agencies, this student-led organisation can help to strengthen learning across the entire medical curricula, to promote the inclusion of student voices in curricula reform, and to ensure that demand for learning on global health is progressively realised. At a local level, there are many examples of students working successfully with faculty members to improve education on global health, as illustrated in the recent Global Doctor publication (13). Our study has helped to highlight the current demand for knowledge on global health from a wide constituency of medical students. Such a demand is only likely to increase along with the broader processes of globalisation, including that of the healthcare workforce. Using a collaborative approach to curricula development that encompasses students’ perspectives, an effective response to learning needs on global health must be prepared and integrated with broader goals for health professionals for the 21st century.

## Summary

Among medical students, there is demand for education on global health, and particularly women's and children's health in low-income countries. The amount, timing, and mode of delivery of existing teaching on these topics currently fail to meet the needs and expectations of medical students. To help address this gap, a collaborative approach is proposed which includes students’ views in the processes for revitalising medical curricula to meet the needs of the 21st century.
